# Supplementary material for: The Association between COVID-19 Pandemic and the Quality of Life of Medical Students in Silesian Voivodeship, Poland
Source: Int J Environ Res Public Health. 2022 Sep 20;19(19):11888. doi: 10.3390/ijerph191911888 (PMC9565595; doi:10.3390/ijerph191911888)
Supplement: Supplementary file 1 [file ijerph-19-11888-s001.zip › ijerph-1900719-supplementary.pdf]

Supplementary Materials

Supplementary S1.

Table S1. Results of post-hoc tests.

	**Overall QoL**	
	**Period before the COVID-19 pandemic (2019/2020)**	**Lockdown period (2020/2021)**
Period before the COVID-19 pandemic (2019/2020)	-	Z=3.22 ;*p*=0.003
Lockdown period (2020/2021)	Z=3.22 ;*p*=0.003	-
The COVID-19 pandemic period after lockdown (2021/2022)	Z=0.26 ;*p*=1.0	Z=3.29 ;*p*=0.002
	Somatic Domain	
	Period before the COVID-19 pandemic (2019/2020)	Lockdown period (2020/2021)
Period before the COVID-19 pandemic (2019/2020)	-	Z=10.25 ;*p*<0.001
Lockdown period (2020/2021)	Z=10.25 ;p<0.001	-
The COVID-19 pandemic period after lockdown (2021/2022)	Z=18.58 ;*p*<0.001	Z=1.28 ;*p*=0.6
	Psychological Domain	
	Period before the COVID-19 pandemic (2019/2020)	Lockdown period (2020/2021)
Period before the COVID-19 pandemic (2019/2020)	-	Z=3.43 ;*p*=0.001
Lockdown period (2020/2021)	Z=3.43 ;*p*=0.001	
The COVID-19 pandemic period after lockdown (2021/2022)	Z=2.07 ;*p*=0.1	Z=2.08 ;*p*=0.1
	Social relationships Domain	
	Period before the COVID-19 pandemic (2019/2020)	Lockdown period (2020/2021)
Period before the COVID-19 pandemic (2019/2020)	-	Z=0.90 ;*p*=1.0
Lockdown period (2020/2021)	Z=0.90 ;*p*=1.0	
The COVID-19 pandemic period after lockdown (2021/2022)	Z=0.83 ;*p*=1.0	Z=1.38 ;*p*=0.4
	Environmental Domain	
	Period before the COVID-19 pandemic (2019/2020)	Lockdown period (2020/2021)
Period before the COVID-19 pandemic (2019/2020)	-	Z=6.21 ;*p*<0.001
Lockdown period (2020/2021)	Z=6.21 ;*p*<0.001	-
The COVID-19 pandemic period after lockdown (2021/2022)	Z=0.16 ;*p*=1.0	Z=5.95 ;*p*<0.001

Supplementary S2.

Table S2. Summary of the WHQOL-BREF domains (scoring after transformation).

Quality of Life Domain	Period before the COVID-19 pandemic (2019/2020) N=560		The COVID-19 pandemic period after lockdown (2021/2022) N=427		Results of U M-W test
	**M (SD)**	**Range (Min-Max)**	**M (SD)**	**Range (Min-Max)**	
Overall QoL	68.9 (18.1)	0.00-100.0	68.6 (17.9)	12.5-100.0	Z=0.24 *p*=0.8
Somatic	43.2 (12.6)	7.1- 75.0	62.8 (15.2)	21.4-96.4	Z=-18.12 *p*<0.001
Psychological	60.8 (13.2)	16.7-95.8	62.2 (16.8)	12.5-100.0	Z=-1.81 *p*=0.07
Social relationships	69.9 (20.4)	8.3-100.0	69.5 (18.6)	0.0-100.0	Z=0.80 *p*=0.4
Environmental	64.0 (13.7)	25.0-96.9	64.4 (12.6)	21.9-93.8	Z=-0.24 *p*=0.8

M, mean; SD, standard deviation; Min, Minimum; Max, Maximum; Z, results of the U Mann-Whitney test; p, statistical significance

Table S3. Results of multivariable linear regression models for the relationship between QoL domains and particular independent variables (N=987).

Independent Variable	Regression Coefficient (95% CI [a])	*p* [b]
Overall QoL (N = 987, $R^{2\,c}$ = 0.11 , p < 0.001[d])		
Current financial situation (1 = poor, 2 = good)	0.18 (0.12, 0.25)	<0.001
Current place of residence during studies at university (1=family home, 2=dormitory/rented flat or room	-0.08 (-0.14, 0.02)	0.005
Current traditional or electronic cigarettes smoking (1 = yes, 2 = no)	0.11 (0.05, 0.18)	<0.001
Frequency of physical activity (1 = high, 2 = low)	-0.14 (-0.20, -0.08)	<0.001
Ever diagnosed chronic disease (1 = yes, 2 = no)	0.15 (0.09, 0.22)	<0.001
Somatic (N = 987, $R^{2\,c}$ = 0.45 , p < 0.001[d])		
Sex (1 = women, 2 = men)	0.05 (0.004, 0.10)	0.03
Current financial situation (1 = poor, 2 = good)	0.05 (0.008, 0.10)	0.02
Hazardous alcohol use (1 = low risk, 2 = hazard use)	-0.05 (-0.10, -0.005)	0.02
Frequency of physical activity (1 = high, 2 = low)	-0.10 (-0.15, -0.05)	<0.0001
Self-declared health status (1 = bad, 2 = good)	0.28 (0.22, 0.33)	<0.001
Research period (1 = before pandemic, 2 = pandemic period)	0.54 (0.53, 0.63)	<0.001
Psychological (N = 987, $R^{2\,c}$ = 0.17 , p < 0.001[d])		
Current financial situation (1 = poor, 2 = good)	0.09 (0.03, 0.15)	0.001
Hazardous alcohol use (1=low risk, 2=hazard use)	-0.07 (-0.13, -0.01)	0.01
Frequency of physical activity (1 = high, 2 = low)	-0.08 (-0.14, -0.02)	0.007
Self-declared health status (1 = bad, 2 = good)	0.35 (0.29, 0.41)	<0.001
Social relationships (N = 987, $R^{2\,c}$ = 0.11, p < 0.001[d])		
Sex (1 = women, 2 = men)	-0.07 (-0.13, -0.01)	0.02
Marital status (1 = in relationship, 2 = single)	-0.22 (-0.28, -0.15)	<0.001
Current financial situation (1 = poor, 2 = good)	0.10 (0.04, 0.17)	<0.001
Self-declared health status (1 = bad, 2 = good)	0.21 (0.15, 0.27)	<0.001
Environmental (N = 987, $R^{2\,c}$ = 0.25, p < 0.001[d])		
Sex (1 = women, 2 = men)	-0.05 (-0.11, -0.003)	0.03
Current financial situation (1 = poor, 2 = good)	0.24 (0.18, 0.29)	<0.001
Current place of residence during studies at university (1 = family home, 2=dormitory/rented flat or room	-0.10 (-0.15, -0.04)	<0.001
Frequency of physical activity (1 = high, 2 = low)	-0.07 (-0.12, -0.01)	0.01

Self-declared health status (1 = bad, 2 = good)	0.36 (0.30, 0.41)	<0.001

[a] CI, Confidence Interval. [b] *p*, significance to the reference group. [c] R^2, determination of the model. [d] *p*, the significance of the multivariable regression model. The reference group was coded as 1.
